# Retrospective Comparative Analysis of Fluoroscopic-Guided Lumbar Puncture in the Routine Prone Versus Lateral Decubitus Position

**DOI:** 10.7759/cureus.18799

**Published:** 2021-10-15

**Authors:** Dmitriy Bakrukov, Zaid Siddique, Rajiv Mangla, Amar Swarnkar

**Affiliations:** 1 Radiology, State University of New York Upstate Medical University, Syracuse, USA; 2 Neuroradiology, State University of New York Upstate Medical University, Syracuse, USA

**Keywords:** fluoroscopic examination, lumbar puncture (lp), prone positioning, lateral decubitus position, opening pressure

## Abstract

Objective: We sought to investigate patient outcomes such as success rate, fluoroscopy time, and radiation dose for fluoroscopic-guided lumbar puncture procedures performed in the prone position versus the lateral decubitus (LD) position.

Methods: Retrospective chart analysis was performed at a single institution from 2013 to 2019. Cases were separated by performance in the prone or lateral decubitus positions. Data collected include patient characteristics, fluoroscopy time, radiation dose (DAP), puncture level, indication, opening pressure, and success rate. Exclusion criteria include trainee participation and procedures where positioning was unspecified. Mean fluoroscopy time, DAP, and procedure success rate were calculated and compared between groups.

Results: Mean fluoroscopy time (min) was 0.97 and 1.07 in the LD and prone groups respectively (p = 0.21). Mean DAP (mGy) was 43.18 and 42.06 in the LD and prone groups respectively (p = 0.38). Success rate was 98.3% and 89.1% in the LD and prone groups respectively (p = 0.04). Room time (minutes) was 64.46 and 77.77 in the LD and prone groups respectively (p = 0.04).

Conclusion: Our study found no statistically significant difference in terms of fluoroscopic time or radiation dose when comparing fluoroscopic-guided lumbar punctures in the prone versus lateral decubitus positions. Further analysis did show a statistically significant increased success rate and a shorter room time for the lateral decubitus position.

## Introduction

Traditionally, lumbar punctures (LPs) have been performed at the bedside using specific anatomical landmarks as guidance for needle insertion. The addition of fluoroscopic guidance to the lumbar puncture procedure has allowed for improved accuracy in the identification of these anatomical landmarks improving our ability to access the subarachnoid space in a safe and efficient manner [1]. The advantages of image guidance have resulted in the fluoroscopic-guided lumbar puncture (FGLP) becoming a highly requested procedure, especially in the setting of a previous failed bedside attempt or when suspicion of subarachnoid hemorrhage requires the risk of traumatic puncture be minimized [2]. Other indications include, but are not limited to, intrathecal chemotherapy, myelography, cerebrospinal fluid (CSF) opening pressure measurement, and therapeutic CSF drainage. Overall the LP procedure has critical applications in the setting of CSF infection, central nervous system (CNS) disorders, and demyelinating disease [3,4]. 

While evidence-based guidelines are lacking for the performance of FGLP, a previously performed study has shown that nearly 90% of all FGLP's are performed in the prone position, with 72% of opening pressures being performed in the prone position as well [5]. The latter finding is important as studies have previously shown that opening pressures may be overestimated in the prone position when compared to the decubitus position [5,6], a finding that has resulted in the majority of neurologists in our institution requesting that all opening pressures be performed in the decubitus position, a trend likely seen across the country.

In our review of the current literature, we failed to discover adequate evidence-based comparisons between the performance of FGLP in the prone position compared to the lateral decubitus (LD) position. Given the previously identified advantage of opening pressure assessment in the lateral decubitus position, as well as potential factors not allowing for the usage of the prone position (i.e., respiratory/surgical equipment, surgical incisions, injury), the ability to perform lumbar punctures in the lateral decubitus position can serve as a great asset. As such, it is the purpose of this study to investigate patient outcomes for procedures performed in the prone position versus the lateral decubitus position in order to determine the feasibility of performing all FGLP’s in the LD position.

## Materials and methods

Patient population

All patients over the age of 18 having FGLP performed at the State University of New York (SUNY) Upstate University Hospital between 6 January 2013 and 12 January 2019 were identified as potential candidates for review. The radiology case information system was searched using the following code “IR Lumbar”. Cases in which the performance of the procedure included the use of a trainee/resident were identified as a potential confounding variable and thus were excluded from the data set. Cases in which the performing radiologist failed to specify the position of the patient were also excluded, except for cases in which a different term was used consistently to portray a specific position (traditional = prone, standard = lateral). The study was reviewed by the institutional review board (IRB) and was exempted from IRB review according to federal regulation (Exemption category #4, Case Number: 1122694-2), informed consent was waived for this retrospective study.

Procedure technique

All FGLP were performed by experienced subspecialized neuroradiologists comfortable in performing LP in both prone and LD positions. The lumbar puncture procedure began after acquiring informed consent from the patient. Procedures were performed in either the prone or the lateral decubitus position using a Fixed C-arm table in the SUNY Upstate University Hospital radiology department. The position in which the procedure was performed was determined by operator preference and oftentimes requested by the referring physician, most often when opening pressures were required. All procedures were performed using the sterile technique. The majority of cases were accessed at the lumbar (L)3/4 level, with some cases utilizing L2/3, L4/5, and L5/sacral (S)1 at the discretion of the attending radiologist after reviewing appropriate imaging.

Local anesthetics and twenty-two-gauge needles were most commonly used with minimal usage of twenty-gauge needles. Intermittent-pulse fluoroscopy was used to identify the appropriate site for the puncture and to monitor the course of the advancing needle during the procedure until the subarachnoid space was entered, confirmed by the reflux of clear CSF, marking the endpoint of fluoroscope use. The procedure report included the fluoroscopy time, fluoroscopic dose, and any known immediate complications from the procedure, of which there were none during this time period.

Data collection

The “anatomic level of procedure”, “fluoroscopy time”, “patient’s age” and "fluoroscopic dose" were all obtained from the procedure note written by the physician performer. Patients' "body mass index" (BMI) was obtained from accessing the patient's record in the electronic health record system. Indications for procedures were obtained from the original order placed by referring provider. Total room time from which the patient entered the room was entered into the electronic health record system by the technologist. Reasons for procedure failure were obtained from the procedure dictation and were stratified into cases with no CSF drainage (needle reached spinal canal with insufficient CSF return), patient intolerance, or pain that resulted in the termination of the procedure.

Statistical analysis

The unpaired t-test was used to determine p-values for radiation dose (DAP), fluoroscopic time, and BMI. 'N-1' chi-square test was used for success rates, level of lumbar puncture, indication. An alpha value of 0.05 was selected. “Success rate” was calculated as the number of successful procedures divided by the total number of procedures for that position. Statistical analysis was performed with SPSS Statistics for Windows, version 25.0 (IBM Corp., Armonk, NY)

## Results

Patient characteristics and outcomes based on positioning

The BMI (kg/m^2^) was not statistically different (p = 0.424) between the prone group 31.95 (95% CI, 29.51 - 34.49) and LD group 33.19 (95% CI, 32.25 - 33.33) (Table 1). The average age of patients in either group was not found to be statistically significant (48.3 years in LD and 48.1 years in prone p=0.981). There were 69 males and 112 females in the LD group and 18 males and 29 females in the prone group (p = 0.454). The indications for FGLP were not statistically significantly different between the two groups (p = 0.542) with “infection”, “inflammation” and “idiopathic intracranial hypertension” being the three most common indications (Table 1).

**Table 1:** Summarized comparison of patient characteristics in LD and prone groups. LD = Lateral Decubitus position; Prone = Prone position; AMS = Altered mental status; SAH = Subarachnoid hemorrhage; IIH = Idiopathic intracranial hypertension; BMI = Body mass index; Multiple = multiple of the above disorders mentioned within the indication for the same order.

Indication	LD	Prone	p-value
AMS	6%	4%	0.542
SAH	3%	6%	
IIH	24%	15%	
Infection	25%	26%	
Inflammation	14%	21%	
Malignancy	11%	15%	
Multiple	17%	13%	
BMI	33.79 (SD= 0.79, 95% CI = 32.25 – 33.33)	31.95 (SD=1.25, 95% CI = 29.51 – 34.49)	0.424
Gender (male: female)	69:112	18:29	0.454
Age (years)	48.3	48.1	0.981

**Figure 1:**
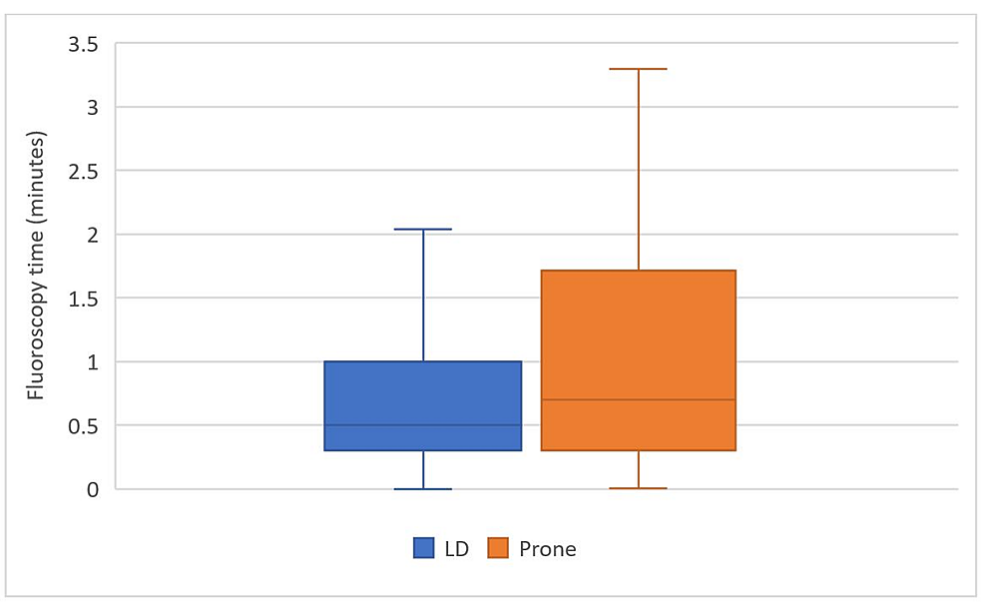
Comparison of total fluoroscopy time (minutes) in LD and prone groups. LD group = Lumbar puncture performed at Lateral Decubitus position; Prone group = Lumbar puncture performed at routine Prone position

**Figure 2:**
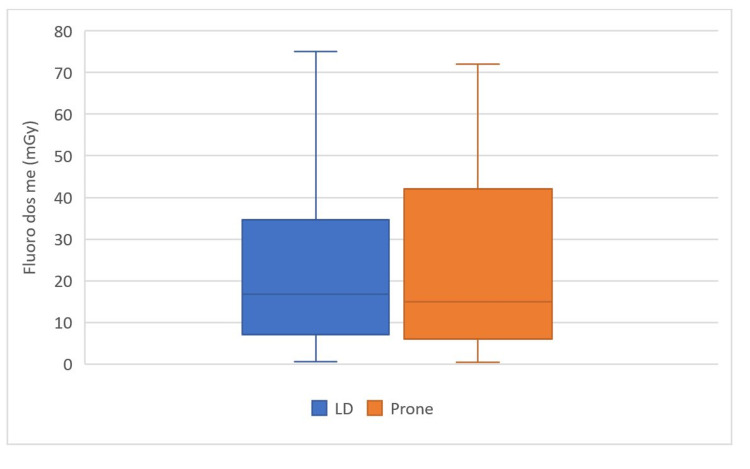
Comparison of total fluoroscopy dose (mGy) in LD and prone groups. LD group = Lumbar puncture performed at Lateral Decubitus position; Prone group = Lumbar puncture performed at routine Prone position

The mean fluoroscopy time (min) was found to be 0.97 (95% CI, 0.76 - 1.18) in the LD group and 1.07 (95% CI, 0.75 - 1.39) in the prone group (p = 0.21) (Figure 1). The mean DAP (mGy) was found to be 43.18 (95% CI, 23.30- 72.68) in the LD group and 42.06 (95% CI, 23.15 - 60.97) in the prone group (p = 0.38) (Figure 2).

The success rate was 98.3% in the LD group and 89.1% in the prone group (p = 0.04) (Table 2). In the LD group, there was a total of six failures out of 181 procedures, and in the prone group, there were five failures out of a total of 46 procedures (Table 2). There was no statistically significant difference in the level at which the puncture was performed (p = 0.060). Opening pressure measurements were obtained in 107 cases in the LD group and 18 cases in the prone group (p = 0.012) with the LD group having a mean opening pressure of 23.4 cmH2O (95% CI, 13.76 - 33.06) and a mean opening pressure of 18.2 cmH2O (95% CI, 10.81 - 25.63) in the prone group (p = 0.014) (Table 2). Room time (min) was 64.46 (95% CI, 32.52 - 96.40) in the LD group and 77.77 (95% CI, 37.60 - 117.94) in the prone group (p = 0.041) (Table 2).

**Table 2:** Summarized Comparison of reported outcomes in the LD and prone groups. DAP = Radiation dose; CSF = Cerebrospinal fluid; L = Lumbar; LD = Lateral Decubitus position; SD = Standard deviation; CI = confidence interval.

Reported Outcome	LD	Prone	p-value
Number	181	46	
Fluoroscopy time (minutes)	0.97 (SD=0.10, 95% CI = 0.76 – 1.18)	1.07 (SD=0.16, 95% CI = 0.75 – 1.39)	0.211
DAP (mGy)	43.18 (SD=12.6, 95% CI = 23.30– 72.68)	42.06 (SD=9.65, 95% CI = 23.15 – 60.97)	0.384
Success Rate	96.70%	89.10%	0.040
				Failures	6	5	0.821
Insufficient CSF	4	3					
Intolerance/Pain	2	2					
Level of puncture (%)			0.060
L2-3	19.1	30.9	
L3-4	69.4	50	
L4-5	11.5	19.1	
Number of opening pressures measured	107	18	0.012
Mean opening pressure (cmH20)	23.4 (SD =0.92, 95% CI = 13.76 - 33.06)	18.2 (SD=1.75, 95% CI =10.81 – 25.63)	0.014
Room Time (minutes)	64.46 (SD=2.92, 95% CI = 32.52 – 96.40)	77.77 (SD=6.60, 95% CI = 37.60 – 117.94)	0.041

## Discussion

Due to recent changes in ordering trends at our institution, our department has seen a significant increase in the number of FGLP orders requesting the procedure be performed in the lateral decubitus, a position that is less commonly used compared to a traditional prone position [5]. Given this trend, the purpose of this retrospective analysis was to compare the outcomes and characteristics of patients undergoing FGLP in the prone and LD positions in order to evaluate the feasibility of performing all lumbar punctures in the lateral decubitus position. In our study, we found there was no statistically significant difference in fluoroscopic dose (43.18 mGy vs 42.06 mGy, p = 0.38) and fluoroscopic time (0.97 vs 1.07 minutes, p =0.21). Interestingly, at our institution, we found a statistically significant higher success rate (96.7% vs 89.1%, p =0.04) and shorter room times (64.46 vs 77.77 minutes, p = 0.04) when comparing the LD vs prone position (see Table 2). Prior studies have demonstrated a higher prone position success rate of 98-99% which would be comparable to our institute's LD position group [6,7]. 

The advantages of the prone approach include operator familiarity, accurate needle trajectory visualization when keeping needle hub and needle tip in line in the center of the screen, as well as being able to use radiography/fluoroscopy (R/F) tables with under-table X-ray tubes which is commonly available and may at times be the only equipment available for the procedure [8,9]. Disadvantages of the prone position include overestimation of opening pressure [10] and potentially narrower interlaminar spaces. The lateral decubitus approach also has its advantages such as positioning the patient with lumbar flexion to improve interlaminar spacing [11], as well as utilization in patients who are unable to tolerate the prone approach (i.e. respiratory/surgical equipment, surgical incisions, injury).

The goal of the performing radiologist was to achieve the greatest technical success while minimizing radiation exposures according to the ALARA (as low as reasonably achievable) principle [12]. Although there are no randomized control trials to evaluate fluoroscopic parameters for prone versus LD positions, conventional radiography principles suggest that there is an increased dose product in the LD position due to an increased distance to the detector and patient dimension necessitating higher exposure times. Adding to this, prior studies have shown a significant difference in the effective patient dose when radiographs are performed in the anteroposterior (AP)/posteroanterior (PA) positions when compared to lateral positions [13]. Additionally, the study by Boddu et al. [4] found that increasing BMI in patients resulted in longer fluoroscopic times when placed in any technical position. When comparing patient population characteristics, we found no statistically significant difference between the LD and prone groups for the following indications: BMI (33.79 vs 31.95, p = 0.42), Gender (69:112 vs 18:29, male:female ratio, p = 0.45) and age (48.3 vs 48.1 years, p = 0.98) (see Table 1). Given that the LD position increases the tissue depth through which the X-ray beam has to penetrate, it could be reasonably inferred that the LD position would result in higher fluoroscopic dosages. The small, but not statistically significant increased fluoroscopic dose observed in the LD position could also be contributed by a slightly higher BMI seen in the LD patients in our study. Prior studies have shown BMI to have an impact on dose [4], likely due to the increased distance from the tube to the detector in patients who measure greater in transverse dimensions than AP.

Previous studies by Cauley et al. have suggested appropriate fluoroscopic times in non-complicated cases should be between 0 - 0.3 minutes. Comparatively, a study by Yang et al. suggests a benchmark of 0.26 minutes for graduating neuroradiology fellows [11]. Additionally, a previous study by Brook et al. found a total average procedure time of 12 minutes with an average effective dose estimate of 2.9 mSv in obese patients with prior failed unguided attempts [6]. Our overall fluoroscopic times and dosages appear to be higher compared to the prior mentioned studies. This may be due to a variety of reasons such as increased BMI in our study, increased anatomic and case complexity.

In our institute, LD compared to the prone group showed higher success rates (98.3% vs 89.1%, p =0.04) and shorter total room times (64.46 min vs 77.77 min, p = 0.04). However, it is important to consider that prior studies have demonstrated a higher prone position success rate of 98-99% which would be comparable to our institute's LD position group [6,7]. The reasons for failure/procedure termination were similar between the two groups (Table 2). We believe the higher success in the LD group at our institute may be due to a variety of factors including operator experience, patient complexity, and potentially due to the previously described advantage of the LD position allowing for increased lumbar flexion to improve interlaminar spacing. We also did not see any significant difference in the number of failures due to patient intolerance between the lateral decubitus and prone positioning groups. The significantly decreased room time seen in the LD group may be a result of a combination of not needing to reposition the patient to obtain lateral decubitus opening pressures, improved CSF flow, and faster access into the CSF space from increased lumbar flexion.

Previous studies by Schwartz et al. [10] and Abel et al. [5] have sought to investigate if measuring opening pressures in the prone vs lateral decubitus positions results in significant differences. In our study, we found a significantly higher average opening pressure in the LD group compared to the prone group (23.4 vs 18.2 cmH20, p = 0.01). However, a significantly higher proportion of patients in the LD compared to the prone group had opening pressures measured and a higher percentage of IIH was seen as an indication for FGLP in the LD group (24% vs 15%, p = 0.54) which may be confounding these findings. Also, potentially not adding needle length to the manometer when in the prone position may have resulted in underestimation of opening pressures in the prone group, a finding which has been seen in prior studies [5]. 

Given the results of a prior survey showing that the vast majority of lumbar punctures are performed in the prone position, we believe that in general there may be a lack of comfort and expertise in the performance of FGLP in the LD position due to inexperience [5]. It is also likely that our proceduralists were not all equally experienced in the LD approach as their training is likely to have focused on the prone approach. Previous studies have shown that fluoroscopic time can improve with training, therefore we expect that over time we may see improvement in patient outcomes such as fluoroscopic dose, time, and success rate as radiologists become more familiar with the LD approach [6,14,15].

We acknowledge that our study was limited due to a variety of factors including but not limited to its retrospective nature, low sample size, and our exclusion of procedures performed by trainees. In both groups, we found a greater than 89% success rate which highlights the fact that FGLP remains an invaluable tool for the safe and consistent access of the CSF space for diagnostic and therapeutic applications in modern radiologic practice. The role of the radiologist for image-guided procedures such as FGLP continues to grow in modern medicine and further analysis into these techniques and benchmarks is vital to improving patient outcomes. We conclude that the utilization of LD positioning in FGLP can be a valuable asset in the practicing radiologist’s arsenal as it can offer some technical advantages for patients who are unable to tolerate prone positioning and who require accurate opening pressures.

## Conclusions

Our study found no statistically significant difference in terms of fluoroscopic time or dosage for lumbar punctures performed in the traditional prone and left lateral decubitus positions. We did find a statistically significant increased success rate and decreased room time for the LD position compared to the prone positioning. Due to recent trends favoring the obtainment of opening pressures in the lateral decubitus position and given the added advantage of greater patient tolerance, we believe a more universally accepted use of FGLP in the LD position is warranted. Radiologists should receive training and get further experience in performing FGLP in the LD position as it can offer some unique advantages.
